# Effect of sitting and lying Liuzijue for lung rehabilitation in acute exacerbation of chronic obstructive pulmonary disease patients with non-invasive ventilation: Study protocol for a randomized controlled trial

**DOI:** 10.1097/MD.0000000000022111

**Published:** 2020-09-18

**Authors:** Jing Yi, Fang Wang, Rensong Yue, Qiao Lin, Ruolan Ding, Xiaohong Xie, Hui Jiang, Feng Jian, Yue Li, Qiurun Zhong

**Affiliations:** a Nursing School, Chengdu University of Traditional Chinese Medicine; b Department of Respiratory Medicine; c Department of Endocrinology, Hospital of Chengdu University of Traditional Chinese Medicine, Chengdu, Sichuan Province, P.R. China.

**Keywords:** acute exacerbation of chronic obstructive pulmonary disease, pulmonary rehabilitation, randomized controlled trial, sitting and lying Liuzijue

## Abstract

**Introduction:**

Chronic obstructive pulmonary disease (COPD) is a lung disease with the highest incidence and high mortality in the world. Acute exacerbation of chronic obstructive pulmonary disease (AECOPD) can significantly accelerate the progression of the disease. Pulmonary rehabilitation is one of the effective treatment methods in COPD patients, but few studies have focused on the effect of pulmonary rehabilitation in AECOPD patients. Liuzijue can improve the pulmonary function and relieve symptoms of COPD patients. However, due to the influence of disease and non-invasive ventilation (NIV), AECOPD patients have poor compliance with getting out of bed at the early stage. Sitting and lying Liuzijue is more suitable in AECOPD patients with NIV. Therefore, this study will evaluate the effect of sitting and lying Liuzijue for lung function, exercise endurance, and quality of life in AEOPD patients with NIV.

**Methods:**

This study is a clinical randomized controlled trial. Sixty four AECOPD patients with NIV will be randomly divided into the experimental group and the control group. All participants will be treated with routine treatment and nursing according to their specific condition. The experimental group will be combined with sitting and lying Liuzijue on the basis of the control group. The duration of the exercise will be 3 months. The primary outcomes are the pulmonary function test and 6-minute walking test (6MWT). The secondary outcome measures include blood gas parameters, dyspnea index (the Modified Medical Research Council Dyspnea Scale [mMRC]), the body-mass, airflow obstruction, dyspnea and exercise capacity (BODE) index, anxiety, and depression (Hospital Anxiety and Depression Scale [HADS]), and quality of life (St·George Respiratory Questionnaire [SGRQ]). The measurement of outcomes will be evaluated at week 13.

**Discussion:**

It's imperative to focus on pulmonary rehabilitation in AECOPD patients. The purpose of this study is to evaluate the effect of sitting and lying Liuzijue for pulmonary rehabilitation in AECOPD patients with NIV.

**Trial registration:**

ChiCTR2000034530, Registered on July 8th, 2020.

## 1 Introduction

Chronic obstructive pulmonary disease (COPD) is a lung disease with the highest incidence in the world.^[1]^ It is estimated that it will be the third leading cause of death, and the economic burden of disease will be the fifth in the world by 2020. The morbidity and mortality of COPD in China are increasing year by year. The results of recent research confirmed for the first time that there are about 100 million COPD patients in China (accounting for about 25% of the total number of COPD patients in the world). The prevalence rate of COPD in people over 40 years old is as high as 13.7%, which is the highest in all countries.^[2]^ In China, the overall disease burden has ranked second. COPD has become the third most common chronic disease after hypertension and diabetes.

Acute exacerbation of chronic obstructive pulmonary disease (AECOPD) is an important course in the clinical process of COPD. It can significantly accelerate the progression of the disease. A study shows that 25% of the decline in lung function of COPD patients can be attributed to AECOPD. The acute exacerbation of COPD could not fully recover their peak pulmonary function flow and health status after 3 months, which seriously affected their work and quality of life.^[3]^

At present, in addition to oxygen therapy and drug therapy, pulmonary rehabilitation is considered to be an effective treatment in COPD patients.^[4]^ For a long time, pulmonary rehabilitation has been used for moderate and severe stable COPD patients. In addition, it is also effective for AECOPD patients after infection controlled and treated with mechanical ventilation.^[5]^ Mohammed et al^[6]^ found moderate and high intensity and continuous aerobic training can not only improve the respiratory function in moderate and severe COPD patient, but also effectively reduce the risk of cardiovascular disease. HE et al^[7]^ selected 66 AECOPD patients to receive pulmonary rehabilitation intervention on the second day of admission until discharge. The intervention group received exercise training (including stretching, endurance strength training) and breathing training (including respiratory control, lip contraction breathing), twice a day, 30 minutes each time. Compared with the baseline, the 6-minute walk test (6MWT) of the intervention group increased by 49 m, and the Brog dyspnea score was significantly improved. Compared with the control group, the Modified Medical Research Council Dyspnea Scale (mMRC) and the body-mass, airflow obstruction, dyspnea and exercise capacity (BODE) index were significantly improved. It is suggested that pulmonary rehabilitation can improve the exercise ability and quality of life of patients. And there are no adverse events in the process of intervention, which confirms the safety of early pulmonary rehabilitation intervention. The 2016 Cochrane evidence-based analysis also shows that pulmonary rehabilitation in AECOPD patients can improve health-related quality of life and exercise endurance.^[8]^

Modern pulmonary rehabilitation program mainly includes: exercise training, breathing training, physical therapy, nutritional support, psychotherapy, health education, and so on. Traditional Chinese medicine (TCM) pulmonary rehabilitation projects mainly include: traditional Chinese medicine, acupuncture, functional exercise, massage, acupoint application, dietotherapy, psychotherapy, and so on. The findings from previous studies suggest that Chinese traditional exercise, including tai chi, liuzijue, wuqinxi, and yijinjing, have positive effect on COPD patients.^[9]^

Liuzijue, a kind of traditional Chinese Qigong, emphasizes the integration of body adjustment, breath adjustment, and heart adjustment in the process of exercise.^[10]^ Liuzijue adopts reverse abdominal breathing, mouth exhale, and nose inhale to match the action. At the same time, emphasizes the role of spirit and ideas. When exhaling, concentrate and think about the viscera corresponding to each word. The 6 sounds of “xu,” “he,” “hu,” “si,” “chui,” and “xi” in Liuzijue correspond to lung, heart, spleen, liver, kidney, and Sanjiao respectively. Respiratory muscles such as intercostal muscle, rectus abdominis muscle, and pectoralis major muscle are mobilized to participate in training so as to deepen respiratory depth and improve pulmonary ventilation.^[11]^ A large number of studies have confirmed that Liuzijue can improve lung function, dyspnea symptoms, exercise endurance, and quality of life in stable COPD patients.^[11–13]^

However, AECOPD patients often have severe dyspnea, respiratory muscle fatigue and respiratory acidosis during hospitalization, so they need to be treated with non-invasive ventilation (NIV).^[14]^ On the other hand, nutrition level and hormone medication will cause patients to feel tired. At the same time, they are also worried that getting out of bed will aggravate their illness.^[15,16]^ Thus AECOPD patients have poor compliance with getting out of bed at the early stage, especially those with NIV. Therefore, it is particularly urgent to explore an effective exercise method suitable for AECOPD patients with NIV. Sitting and lying Liuzijue is a kind of medium and low intensity aerobic exercise which is on the basis of Liuzijue. It is suitable for people who are too old and frail to stand for a long time. Compared with the standing Liuzijue, it is more appropriate for long-term bedridden AECOPD patients with NIV.

Therefore, in this study, sitting and lying Liuzijue will be used to carry out early pulmonary rehabilitation exercise in AECOPD patients with NIV. The purpose of this study is to evaluate the effect of sitting and lying Liuzijue on blood gas index, pulmonary function, dyspnea symptoms, exercise endurance, BODE index, anxiety and depression, and quality of life. Thus, to provide a safe, effective, and good compliance method of TCM pulmonary rehabilitation exercise for AECOPD patients with NIV.

## 2 Methods/design

### 2.1 Design

**Figure 1:**
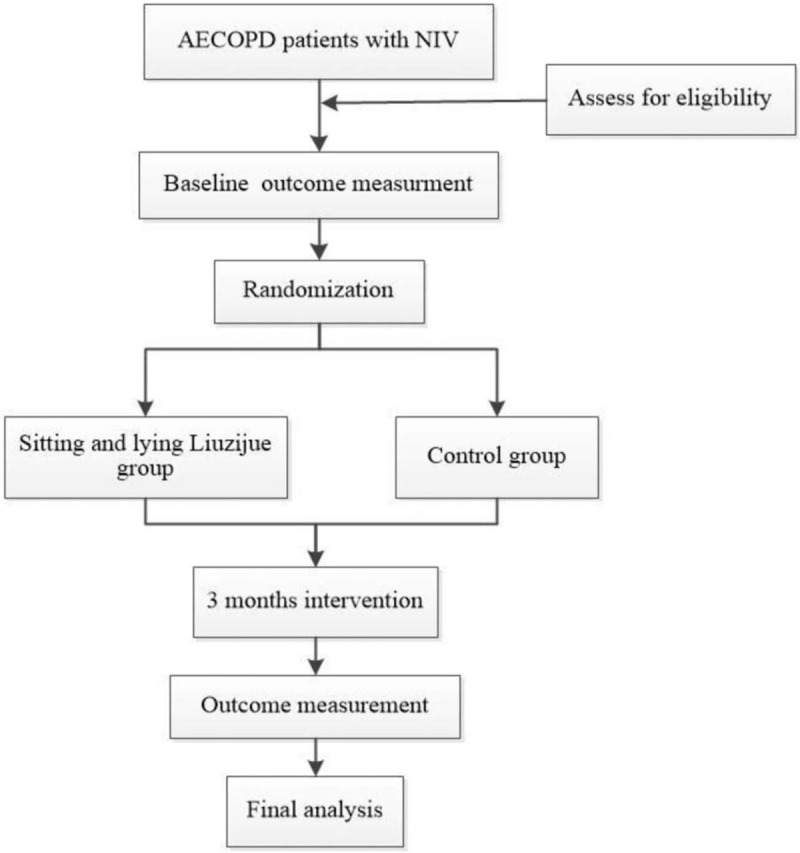
Study procedure.

**Figure 2:**
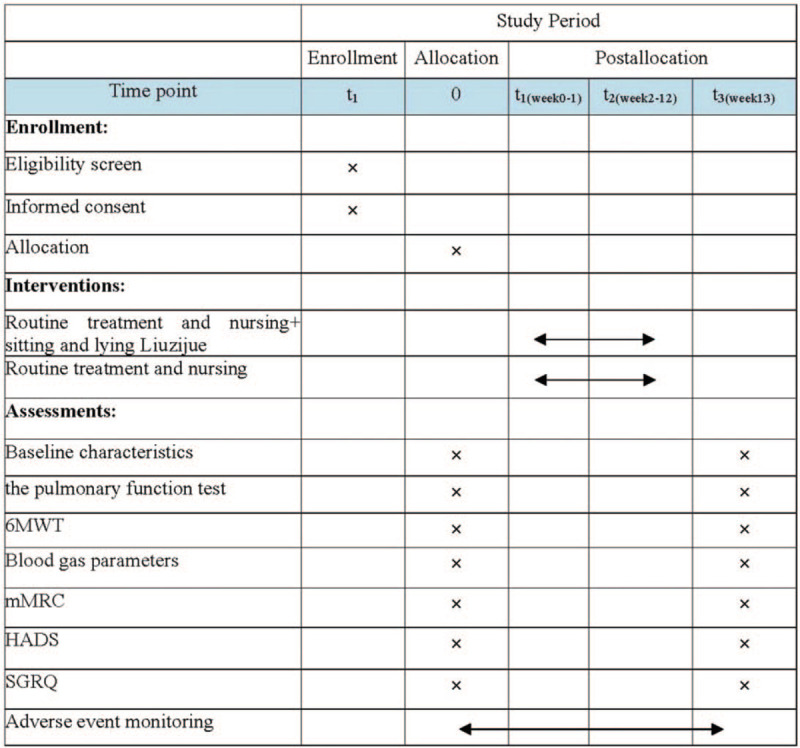
Illustration of the design for clinical studies. 6MWT = 6-minute walking test, HADS = Hospital Anxiety and Depression Scale, mMRC = the Modified Medical Research Council Dyspnea Scale, SGRQ = St·George Respiratory Questionnaire.

This study is a clinical randomized controlled trial. Sixty four AECOPD participants with NIV will be conducted in the Hospital of Chengdu University of TCM (Chengdu, China). Participants will be recruited through a recommendation by the respiratory clinician. The participants will be informed of the benefits and risks of the study in detail. After obtaining written informed consent, eligible participants will be randomly assigned to the experimental group and control group in a ratio of 1:1. To prevent design bias, we will follow the Consolidated Standards of Reporting Trials statement^[17]^ and the Standard Protocol Items: Recommendations for Interventional Trials (SPIRIT) 2013 statement.^[18]^ The study procedure is shown in Fig. 1 and the time points of the study is shown in Fig. 2.

### 2.2 Ethics approval

The study is in compliance with the Declaration of Helsinki (Edinburgh 2000 version). This study was reviewed by the Ethics Committee of the affiliated Hospital of Chengdu University of traditional Chinese Medicine (Ethics No: 2019KL-064), following the principles of voluntariness, impartiality, advantage, and confidentiality. Explain the purpose, scheme, intervention time, benefits, and potential risks of this study to patients before intervention. Strictly abide by the wishes of the patients, agree and sign the informed consent form before entering the research group. In the process of intervention, patients have the right to withdraw from this study according to their own conditions. Strictly implement the termination standard to ensure the safety of patients. Personal information and related records of patients will be kept strictly confidential. We registered the study in the Chinese Clinical Trial Registry (Registration number: ChiCTR2000034530) in 2020. If there is any amendment to the protocol, approval must be again sought from the Ethics Committee.

### 2.3 Sample size

The calculation of sample size is based on the main results (6MWT). According to the previous literature,^[19]^ it is estimated that the average score of the control group was 442.5 and the standard deviation was 61.5. After Taiji Qigong exercise, the average of the experimental group was 486.7 and the standard deviation was 67.3. formula for calculating the sample size is as follows: 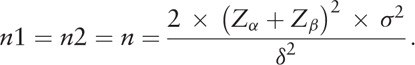


At the 5% significance level, a total of 26 patients are needed in each group to achieve 80% power. The estimated dropout rate is 20%, and a total of 32 patients have been included in each group.

### 2.4 Participants

#### 2.4.1 Diagnostic criteria

1. The diagnostic criteria of COPD are as follows: Refer to the guidelines for the diagnosis and treatment of chronic obstructive Pulmonary Disease (revised in 2013) and the global initiative for chronic obstructive pulmonary disease (GOLD) in 2019.
2. The diagnostic criteria of AECOPD are as follows: Patients with acute exacerbation of respiratory symptoms (typically characterized by increased dyspnea, increased cough, increased sputum and/or purulent sputum), beyond daily variation, and led to the need to change the drug treatment regimen.
3. The standards of NIV are as follows: Respiratory acidosis (arterial blood pH ≤7.35 and/or PaCO_2_ >45 mmHg). Severe dyspnea combined with clinical symptoms. Increased respiratory function, such as the application of auxiliary respiratory muscle breathing, chest-abdominal contradictory exercise, or costal space muscle contraction.

#### 2.4.2 Inclusion criteria

1. Patients with COPD diagnosed according to GOLD.
2. AECOPD patients with NIV.
3. Age range 40 to 80 years.
4. Clear consciousness for communicating normally and completing the self-rating scale.
5. Agree to sign the informed consent and voluntary participation.

Only those who conform to all of the above criteria will be included.

#### 2.4.3 Exclusion criteria

1. Patients with chronic cough and asthma caused by lung cancer, pulmonary interstitial disease, bronchiectasis, bronchial asthma, and other non-chronic obstructive pulmonary diseases.
2. Intolerant to exercise (complicated with severe diseases of cardio-cerebrovascular, liver, kidney, lung and hematopoietic system).
3. Patients with functional impairment of low back and limbs or after operation.
4. Patients with mental illness or speech disorder to communicate difficultly.
5. Patients who are currently participating in other clinical studies or doing other functional exercises.

Those who conform to any of the above criteria will be excluded.

#### 2.4.4 The termination and withdrawal criteria

1. Voluntary withdrawal from the trial or loss of follow-up.
2. Poor compliance.
3. Emergency attack of serious illness during the study and need other treatment.

### 2.5 Randomization

According to the random number table generated by the computer, the participants will be randomly assigned to the 2 groups. The randomized allocation sequence will be performed by a researcher who will not be involved in the recruitment, evaluation, or training of patient. Random numbers will be placed in sealed opaque envelopes to blind the group allocation.

## 3 Interventions

All participants will be treated with routine treatment and nursing according to their specific condition. The experimental group will be combined with sitting and lying Liuzijue on the basis of the control group.

### 3.1 The control group

Participants in the control group will be treated with standard drugs of AECOPD, including bronchodilator (short-acting β 2 agonist and anticholine), oral prednisone (40 mg/d, for 5 days), and antibiotics (amoxicillin-clavulanic acid, respiratory quinolone, macrolides) according to the results of sputum culture if necessary. The range of oxygen concentration in NIV needs to be controlled. It's necessary to adjust the oxygen concentration according to the patient's tolerance and blood oxygen saturation. In addition, oxygen saturation need to be maintained at 90% to 94% for avoiding the occurrence or aggravation of CO_2_ retention and/or respiratory acidosis. Routine respiratory function exercise including lip contraction breathing and abdominal breathing. The routine nursing including many aspects, such as condition observation, respiratory care (pay attention to sputum drainage, active expectoration, etc), diet nursing (eating high-quality protein, high vitamins, low-fat diet, more light and digestible food, more water, and supplement essential diet or high intravenous nutrition who cannot eat anything), psychological nursing (giving psychological comfort and spiritual support) and health education, etc.

### 3.2 The experimental group

On the basis of the control group, participants in the experimental group will practice sitting and lying Liuzijue once every morning and evening, 30 minutes each time, for a total of 3 months. Before the official start of the experiment, 2 members of the research group need to be trained in skills and methods. After successfully passing the examination, they would conduct action guidance and training to the participants. The teaching time of patients should be controlled within 5 days, and they should be assessed on the last day. After the patients have correctly mastered the steps, essentials and matters needing attention of sitting and lying Liuzijue, they will formally enter the experiment.

Sitting and lying Liuzijue exercise begin immediately after the appearance of the lung infection control window. Exercise once every morning and evening, each time for 30 minutes, for a total of 3 months. In the process of exercise, the participants need to take off the NIV mask and use nasal catheters to inhale oxygen. The pulse oxygen saturation meter will be used to closely monitor the changes of heart rate and fingertip oxygen saturation. Take continuous exercise and intermittent exercise according to the tolerance of participants. The next day, ask the participants about their physical recovery and observe their breathing and heart rate during the entire exercise to confirm whether the current exercise intensity is appropriate.

**Figure 3:**
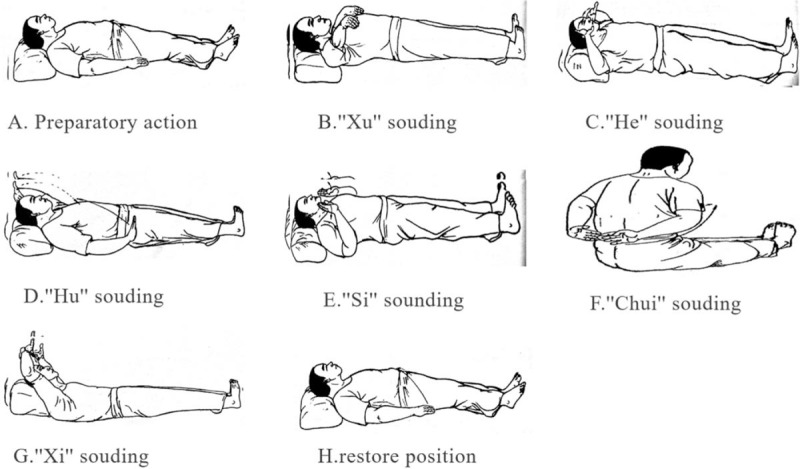
Main characteristics of sitting and lying Liuzijue.

All participants use reverse abdominal breathing, exhale first and then inhale. Read when they exhale, lift the anus and contract the kidney at the same time (abdomen and buttocks, urethral orifice, and anal micro-lift). When inhaling, the lips are gently closed, the tongue is against the upper jaw, the whole body is relaxed, the lower abdomen is naturally raised, and the air is naturally inhaled. After reading each word 6 times, using natural breathing, tongue against the palate (cis-abdominal breathing can also be used). The function is to adjust breathing, restore nature, and take a short rest. The detailed steps are shown in Fig. 3.

When the participant is discharged from the hospital, we will distribute sitting and lying Liuzijue exercise manual, video, and intervention diary. At the same time, communicate, guide, and supervise through telephone and WeChat groups.

### 3.3 Compliance

Keep communication effectively with participants and establish a good relationship. Inform participants the importance of practicing sitting and lying Liuzijue for disease recovery. During hospitalization, members of the research group will guide and supervise participants every day. When the participants are discharged from hospital, distribute “intervention diary” and conduct guidance and supervision through telephone and WeChat groups. If the participants are not motivated enough during the trial, they would be encouraged and the frequency of supervision would be increased. In the process of intervention, participants can share and communicate with each other through WeChat groups for promoting compliance.

### 3.4 Outcome measures

The primary outcomes are the pulmonary function test (FEV_1_%, FEV_1_/FVC, and FVC) and 6-minute walking test (6MWT). The secondary outcome measures include blood gas parameters (PH, PaCO2, PaO2), the mMRC, Hospital Anxiety and Depression Scale (HADS), and St·George Respiratory Questionnaire (SGRQ). Baseline data will be collected before intervention. Outcome indicators will be collected at the 13th week.

The measurement of pulmonary function will be carried out by the same pulmonary function technician in the respiratory department. Before the test, the participants need to take a rest quietly for 10 minutes, then inhaled the bronchodilator salbutamol 200 μg. The arterial blood will be collected by the nurses in the department for blood gas analysis. 6MWT will be carried out by trained team members. Before the test, the participants need to take a rest for 10 minutes, and their blood pressure, pulse, and blood oxygen saturation will be measured. Carry a portable pedometer and walk as fast as possible. At the end of time, blood pressure, pulse, and blood oxygen saturation will be measured again, and the walking distance will be recorded. All the scales involved in this study will be completed by 3 specialized researchers who had received professional training and passed the examination.

### 3.5 Adverse events

During the trial, the adverse reactions such as palpitations, sweating, pallor, dyspnea, and blood oxygen saturation lower than 85% will be accurately recorded. Once there are adverse reactions, patients should be stopped immediately and have a bed rest. Researchers need to observe closely the condition, and report to the doctor in time. The doctor will judge the degree of the adverse events initially and its correlation with the experimental intervention, thus give further treatment suggestions. At the same time, researchers should also make detailed records, such as the degree, time, treatment measures, and outcome of adverse events.

### 3.6 Statistical analysis

Two researchers who are not be involved in the recruitment, evaluation, or training of patient will be responsible for entering the data. Statistical analysis will be carried out by using SPSS 24.0 statistical software. If the measurement data accorded with normal distribution, it will be described by mean and standard deviation. If not, it will be described by median and quartile distance. The measurement data will be examined using group *t* tests or non-parametric tests, the count data will be tested using a Chi-square test or Fisher exact probability method, and the grade data will be tested using non-parametric tests. All statistical tests will be bilateral tests and *P* < .05 will be considered to indicate statistical significance.

## 4 Discussion

AECOPD is an important part of the course of COPD. AECOPD can lead to a sharp decline in lung function, skeletal muscle strength, negative nutritional imbalance, and other symptoms. It seriously affects the quality of participants’ life and causes serious economic burden.^[20]^ It is necessary to provide a simple, safe, effective, and compliant pulmonary rehabilitation method for AECOPD participants with NIV. Under the guidance of the theory of TCM, sitting and lying Liuzijue is to adjust and control the rise and fall of breath in the body through the specific 6-word vomiting training of “xu,” “he,” “hu,” “si,” “chui,” and “xi.” Thus improve the viscera functions of the human body, such as liver, heart, spleen, lung, kidney, Sanjiao, and maintain the balance of the whole body.^[11]^ It is one of the applicable technologies of TCM designated and promoted by the State Administration of TCM. Sitting and lying Liuzijue is safe, effective, and high compliance, which is easy for participants to accept. Studies have shown that participants in the stable phase of COPD can carry out long-term exercise, the application prospect is good.^[10–13]^

At present, the ways of pulmonary rehabilitation exercise generally include aerobic exercise, resistance exercise, aerobic combined with resistance exercise.^[21]^ Training can only be done with instruments. This is not suitable for China's national conditions. For the lung rehabilitation of stable COPD patients, Chinese traditional exercise is mostly used in domestic research. Such as Baduanjin, Taijiquan, Wuqinxi, and Liuzijue.^[13,22,23]^ However, there are few reports of traditional exercise intervention for AECOPD patients. There is no report of pulmonary rehabilitation intervention in AECOPD patients with NIV by using sitting and lying Liuzijue. Therefore, we designed this clinical randomized controlled trial to evaluate the effect of sitting and lying Liuzijue in AECOPD patients with NIV.

However, it can not be ignored that this study also has some limitations. First of all, the study is a kind of sports intervention. It is difficult to achieve the blind method of implementers and participants, so there may be a placebo effect leading to deviation. Secondly, sitting and lying Liuzijue is a kind of traditional Chinese exercise, so the cognition of it will be limited by geography, which may hinder its application and promotion. Finally, the participants in the study will be all from the same hospital in Sichuan, China. It's not clear whether the relative effects of the sport in other ethnic groups are similar. However, despite the limitations, we believe that this study is expected to confirm that sitting and lying Liuzijue can improve lung function, exercise endurance, quality of life, and create better social benefits in AECOPD patients with NIV.

## Acknowledgments

The authors are grateful to the Sichuan Science and Technology Planning Project for funding this study.

## Author contributions

**Conceptualization:** Jing Yi, Qiao Lin, Hui Jiang.

**Funding acquisition:** Fang Wang.

**Investigation:** Xiaohong Xie, Feng Jian, Yue Li, Qiurun Zhong.

**Supervision:** Ruolan Ding, Fang Wang, Rensong Yue.

**Writing – original draft:** Jing Yi.

**Writing – review & editing:** Fang Wang.

## Abbreviations

Abbreviations: 6MWT = 6-minute walk test, AECOPD = acute exacerbation of chronic obstructive pulmonary disease, BODE = the body-mass, airflow obstruction, dyspnea and exercise capacity, COPD = chronic obstructive pulmonary disease, FEV_1_ = forced expiratory volume in 1 second, FVC = forced vital capacity, GOLD = the global initiative for chronic obstructive lung disease, HADS = Hospital Anxiety and Depression Scale, mMRC = the Modified Medical Research Council Dyspnea Scale, NIV = non-invasive ventilation, PASS = Power Analysis and Sample Size, SGRQ = St·George Respiratory Questionnaire, SPIRIT = Standard Protocol Items: Recommendations for Interventional Trials, TCM = traditional Chinese medicine.
